# Multiparametric Flow Cytometry-Based Immunophenotyping of Mouse Liver Immune Cells

**DOI:** 10.3390/mps5050070

**Published:** 2022-09-03

**Authors:** Lenka Vanekova, Marketa Pimkova Polidarova, Vaclav Veverka, Gabriel Birkus, Andrea Brazdova

**Affiliations:** 1 Institute of Organic Chemistry and Biochemistry of the Czech Academy of Sciences, Flemingovo namesti 542/2, 160 00 Prague, Czech Republic; 2 Department of Cell Biology, Faculty of Science, Charles University, Vinicna 7, 128 00 Prague, Czech Republic

**Keywords:** flow cytometry, immunophenotyping, mouse liver, PBS-based liver perfusion, non-parenchymal cells

## Abstract

The liver is a complex organ that governs many types of metabolisms, including energy metabolism and other cellular processes. The liver also plays a crucial role in important functions in immunity, and the activity of liver tissue-associated immunity affects the outcome of many liver pathologies. A thorough characterization of the liver immune microenvironment may contribute to a better understanding of immune signaling, the mechanisms of specific immune responses, and even to improved predictions about therapy outcomes. In this paper, we present an optimized, simple, and rapid protocol to characterize the liver-associated immune cell milieu. We believe that the most suitable technique for obtaining a complex immune cell suspension and for removing contaminating blood cells is to perform mouse liver perfusion, using only phosphate buffer saline. Combining an enzymatic digestion and a mechanical dissociation of liver tissue, followed by cell purification, improves downstream applications. This combination is an essential prerequisite for immune cell determination and characterization. We then demonstrate a flow cytometry-based multiparametric immunophenotyping along with a gating strategy to detect and quantify liver endothelial cells, T cells (helper and cytotoxic), B cells, NK cells, NKT cells, neutrophils, monocytes (subsets included), dendritic cells (subsets included), macrophages and Kupffer cells.

## 1. Introduction

The liver is a complex organ, consisting of multiple cell types. The majority of them, 60–80%, are the parenchymal cells—hepatocytes. The remaining cells form a heterogeneous population of non-parenchymal cells (NPC), primarily composed of liver endothelial cells (LEC), hepatic stellate cells (HSC) and immune cells [1,2,3]. Intrahepatic immune cells are the most prominent component (up to 50%) of NPC [4], consisting of T cells (both cluster of differentiation (CD) 8+ and CD4+), B cells, natural killer (NK) and natural killer T (NKT) cells, neutrophils, monocytes and various subtypes of dendritic cells (DCs) and macrophages [2,3,5]. The relative composition of the liver immune cells varies depending on the physiologic conditions and species, e.g., the NKT cells are more abundant in the liver of mice than humans [2]. Liver immune cell composition differs from lymphoid organs or blood, as the liver is enriched with CD4+ T cells, NK, NKT and gamma delta (γδ) T cells [2,6]. In general, liver is mostly tolerogenic organ, which is important to prevent inflammatory responses to diet and intestinal microflora [7]. As such, NPC actively contribute to the immune tolerance [7], e.g., hepatocytes, Kupffer cells (KC) and LEC induce anergy of T cells [8,9]. In addition, sinusoidal LEC (LSEC) produce lectin called LSECtin that triggers CD8+ T cell tolerance [10]. Moreover, KC secrete the anti-inflammatory cytokine interleukin 10 (IL10) [11], and the liver-resident NK cells can suppress T cells via interaction with immune check-point pathways [12]. Under certain pathological conditions such as infection, an inflammatory disorder, or cancer, the liver immune cell distribution or function may change. This can lead to a potential imbalance and alteration of immune cell crosstalk [1,2,5,13,14]. To better understand various pathologies affecting the liver, the resident/infiltrating immune populations need to be isolated, immunophenotyped and quantified. The goal is to obtain a single cell suspension of high yield, while still preserving antigen/epitope profiles (minimum epitope degradation) and retaining cell viability for subsequent downstream applications [4,15,16,17,18,19,20,21]. In order to avoid cross-contamination of the liver residing in immune subsets from those found in the blood, the in situ perfusion of the liver is performed via the vena cava or the portal vein [22]. The next crucial step is the proper processing of the liver tissue. The tissue dissociation can be performed either by a mechanical disruption, by an enzymatic digestion using a collagenase, or a combination of both approaches [21,22,23]. For liver immune cell phenotyping, the hepatocytes need to be removed from the obtained homogenate, as they may interfere with later downstream immune profiling [18,19]. The cell suspension can be further purified, either by multiple centrifugation steps [3,18] or by purification through a Percoll or Iodixanol density gradient [4,18,19,20,21,23]. However, this procedure is time and material consuming. In addition, the harsh conditions of the purification methods may also affect the viability and/or function of cells intended for any downstream application. After the isolation of NPC, the cells can be further analyzed and/or cultivated. Although several studies have focused on the characterization of a single or a few populations, such as macrophages [24], KC [18,19], LEC [17,18,22] or NK cells [16], we and others [21,25] aim to analyze multiple immune populations. Several immunophenotyping methods are available. Conventional or spectral flow cytometry is often used for various immunophenotyping [26,27]. Different approaches are cytometry by time-of-flight (CyTOF), in which mass spectrometry analysis of single cells labeled with isotope-conjugated markers is used [15], or automated parallel RNA single-cell sequencing combining fluorescence-activated cell sorting techniques [28] and massive multiplexing RNA sequencing [29]. Although CyTOF and an automated massively parallel single-cell RNA sequencing approach allow analysis of more than 20 colors in one panel, the costs and instrumental setup, both make it rarely available in regular academic laboratory conditions.

In the presented protocol, we describe a robust yet low-cost, fast, effective, practical, and straightforward procedure for the isolation of mouse liver NPC that relies on a mouse liver dissociation kit from Miltenyi Biotec [30]. Moreover, we present a thorough immunophenotyping protocol using conventional flow cytometry that allows for the detection and quantification of various immune populations in one single sample. To specifically analyze hepatic immune microenvironment avoiding red blood cell contamination, the procedure consists of liver perfusion with PBS, liver tissue dissociation by combining mechanical disruption and enzymatic digestion, followed by the purification of cells and immunophenotyping. We address a multiparametric flow cytometry analysis, valuable for both regular and large-scale screenings, including a gating strategy to detect and quantify LEC, T cells (helper and cytotoxic), B cells, NK cells, NKT cells, neutrophils, monocytes (reparative and inflammatory), DCs (including their subsets), macrophages and KC. The method can be useful in research focusing on the characterization of the liver immune milieu in mouse models of human pathologies, or in studies of the liver immune response to different treatments. This method could also be valuable for regular small as well as large-scale screenings, e.g., a preclinical evaluation of drug efficacy.

## 2. Experimental Design

### 2.1. Materials

1. Debris removal solution (Miltenyi Biotec, Bergisch Gladbach, Germany; Cat. no.: 130-109-398; store protected from light at 4 °C, do not freeze)
2. DMEM High Glucose w/stable glutamine, w/sodium pyruvate (Biowest, Riverside, MO, USA; Cat. no.: L0103-500; store protected from light at 4 °C, do not freeze)
3. Aerrane (Isoflurane UPC, Baxter, Deerfield, IL, USA; Cat. no.: FDG9623; store protected from light at room temperature (RT))
4. Liver dissociation kit, mouse (Miltenyi Biotec, Bergisch Gladbach, Germany; Cat. no.: 130-105-807; individual components store at 4 °C, reconstituted components store for max. 6 months at −20 °C, avoid freeze/thaw cycles)
5. Phosphate buffered saline w/o calcium, w/o magnesium (Biowest, Riverside, MO, USA; Cat. no.: P0750; store at 4 °C)
6. Red blood cell lysis buffer (RBL; store at RT; see Reagent Setup)
7. Trypan blue solution (Sigma-Aldrich, Burlington, MA, USA; Cat. no.: T8154, store at RT)
8. Fixation buffer (BD Biosciences, Franklin Lakes, NJ, USA; Cat. no.: 554655, store protected from light at 4 °C, do not freeze), optional reagent
9. Flow cytometry (FC) staining buffer (store for max. 1 month at 4 °C, do not freeze; see Reagent Setup)
10. Fluorescently labeled antibodies for FC purposes (see Table 1; store protected from light at 4 °C)

### 2.2. Equipment

- 25 mL tissue sample vessel (Carl Roth, Karlsruhe, Germany; Cat. no.: AYX2.1)
- Blunt dissecting scissors (VWR^®^, Radnor, PA, USA; Cat. no.: HAMMHSB120-14)
- Cotton pads (Batist Medical a.s., Cerveny Kostelec, Czech Republic; Cat. no.: 5670)
- Dry bath incubator (Major Science, Saratoga, CA, USA; Cat. no.: MD-02N)
- 15 mL conical centrifuge tubes (VWR^®^, Radnor, PA, USA; Cat. no.: 525-1084)
- 1.5 mL microcentrifuge tubes (VWR^®^, Radnor, PA, USA; Cat. no.: 89000-028)
- Forceps with round blade (VWR^®^, Radnor, PA, USA; Cat. no.: 232-0106)
- Forceps with straight blade (VWR^®^, Radnor, PA, USA; Cat. no.: BSNC00DSA)
- gentleMACS C-tube (Miltenyi Biotec, Bergisch Gladbach, Germany; Cat. no.: 130-093-237)
- gentleMACS Octo dissociator with heaters (Miltenyi Biotec, Bergisch Gladbach, Germany; Cat. no.: 130-096-427)
- Ismatec IPC pump (Ismatec, Wertheim, Germany; Cat. no.: ISM 930)
- Luna-II automated cell counter (Logos Biosystems, Anyang-si, Gyeonggi-do, Korea; Cat. no.: L40002)
- Cell counting slides (Logos Biosystems, Anyang-si, Gyeonggi-do, Korea; Cat. no.: L12003)
- MACS SmartStrainer, 100 µm (Miltenyi Biotec, Bergisch Gladbach, Germany; Cat. no.: 130-098-463)
- Neo Delta Ven T cannula 24G (Delta Med, Viadana, Lombardia, Italy; Cat. no.: 3113122)
- R540 Enhanced Anesthesia Machine (RWD, Baltimore, MD, USA; Cat. no.: R540IE)
- Refrigerated centrifuge with swinging buckets (ThermoFisher Scientific, Waltham, MA, USA; Cat. no.: 15253457)
- Sharp scissors (VWR^®^, Radnor, PA, USA; Cat. no.: 233-1104)
- Extension tubes (Gama group, Ceske Budejovice, Czech Republic; Cat. no.: 606301-ND)
- Pump tubing (Tygon^®^, Ismatec, Wertheim, Germany; Cat. no.: ISMCSC0024T, ISMCSC0048T)
- Tweezers (VWR^®^, Radnor, PA, USA; Cat. no.: 229-0374)
- Water bath (Polysciences, Warrington, PA, USA; Cat. no.: WBE20A12E)
- BD LSRFortessa flow cytometer (BD Biosciences, Franklin Lakes, NJ, USA) or any multiparametric flow cytometer with at least 13-fluorescence detectors)

### 2.3. Software

- Diva software (Becton Dickinson, Franklin Lakes, NJ, USA, v8.0.1. or later, BD FACSDiva™ Software www.bdbiosciences.com, accessed on 4 August 2022) or any equivalent
- FlowJo analysis software (BD Biosciences, Franklin Lakes, NJ, USA, v10 or later, www.bdbiosciences.com, accessed on 4 August 2022) or any equivalent

## 3. Procedure

**Figure 1:**
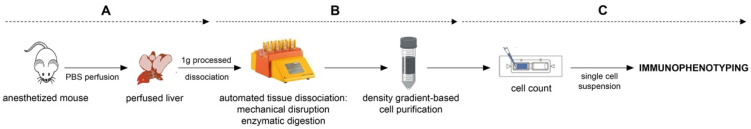
Workflow as a schematic description. (**A**) in vivo manipulation part. (**B**) liver processing. (**C**) downstream procedure.

**Figure 2:**
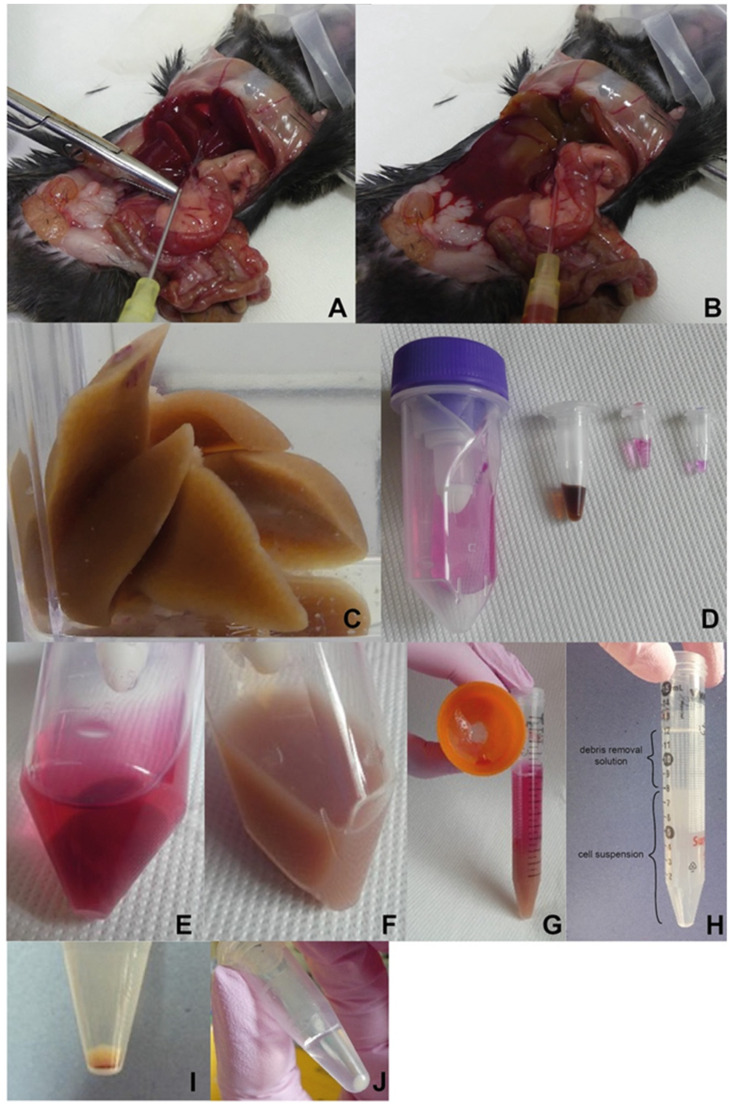
Illustrated work procedure. (**A**) Uncovered and stretched portal vein to perform perfusion. (**B**) Gradual liver perfusion. (**C**) Perfused liver. (**D**) Material preparation for dissociation. C-tube with cell culture medium, dissolved particular components of the liver dissociation kit (all Miltenyi Biotec, described from the left). (**E**) Liver placed into C-tube with the dissociation mix. (**F**) Liver homogenate after dissociation. (**G**) Filtrate of dissociated tissue. (**H**) Density gradient-based cell purification (layer of debris removal solution on top of the cell suspension; before centrifugation). (**I**) Obtained cells contaminated with leftover of red blood cells. (**J**) Final NPC yield after the removal of red blood cells.

**Figure 3:**
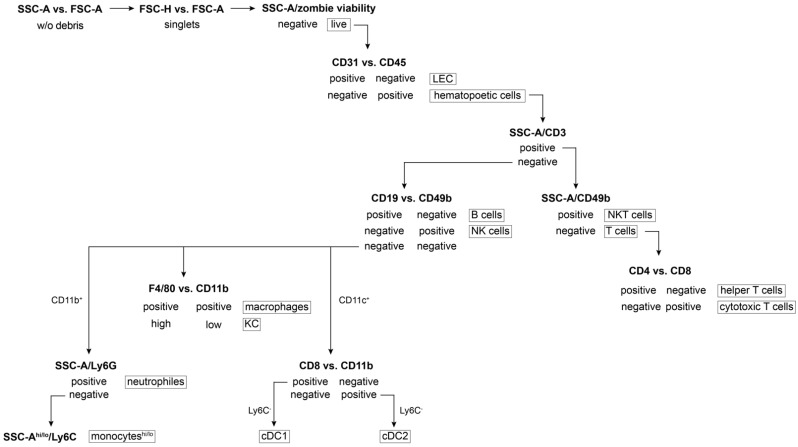
Schema of gating strategy for flow cytometry data acquisition. SSC-A: side scatter-area; FSC-A/H: forward scatter-area/height; hi/lo: high/low population.

**Note**: In this protocol (Figure 1, Figure 2 and Figure 3), we perform a PBS-based perfusion via portal vein using C3H/HeN mice. However, the same method can be applied on any mouse strain. We process the mouse liver by combining a mechanical disruption and an enzymatic digestion. NPC are then purified based on a gradient centrifugation.

### 3.1. Liver Perfusion

1. Set up of instruments: prime peristaltic pump with tempered PBS, set the flow rate to 2.5 mL/min.
2. Anesthetize a mouse using 2–5% isoflurane in air or oxygen mixture until the deep loss of sensitivity.
3. Place the fully anesthetized mouse to a supine position in a breathing mask, attach paws to the pad to stretch the mouse.
4. Disinfect the abdomen with 70% ethanol. Lift the skin with tweezers. Using blunt dissecting scissors, cut the skin and peritoneum horizontally in the lower abdomen. Continue with a lateral cut on both sides of the abdomen, up to the lower rib cage.
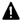
**CRITICAL STEP** Continuously observe breathing rate to be low and deep without any sign of choking. Be sure not to cut any of the organs or diaphragm.
5. Use forceps to grab the abdominal skin and peritoneum and roll the skin up to the rib cage to reveal the abdominal cavity. Move intestines to the side to expose the portal vein.
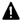
**CRITICAL STEP** For optimal procedure, no bleeding should occur.
6. Straighten the vein. Place the needle of the cannula in parallel to the portal vein with the bevel up (Figure 2A). Inject the lower part of the vein with the cannula needle, then pull out the needle from the cannula, and move the polymer part further into the vein. A blood backflow should be visible.
  **Note**: It is not necessary to immobilize the cannula by a vein ligation.
7. Adjust the pump flow rate to 2.5 mL/min, ensure there are no bubbles in the tubing, place the tubing into the cannula, cut one kidney, and immobilize the tubing onto the pad.
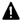
**CRITICAL STEP** Observe an immediate liver color change as a proof of correct perfusion setting (Figure 2B).
8. Wash the liver with 30–40 mL of PBS until the liver completely lightens and no blood appears in the wash volume.
9. Remove the cannula and turn off the pump. Carefully remove the gallbladder and harvest the liver into 50 mL sample vial with PBS (Figure 2C).
10. **OPTIONAL STEP** Carefully remove the gallbladder to prevent bile contamination of the liver; if contaminated, thoroughly wash the liver with PBS.

### 3.2. Liver Dissociation

**Note**: The protocol below does not differ from the manufacturer’s instructions (liver dissociation kit from Miltenyi Biotec [30]).

1. Cut off 1 g of liver tissue (weight should not exceed 1.2 g of tissue per one dissociation [30]).
2. Wash the liver with preheated cell medium and place it into C-tube with liver dissociation mix (according to the manufacturer’s instruction). After attaching C-tube onto the dissociator with heater, launch a 37C_m_LIDK_1 program predefined by the manufacturer (Figure 2D,E).
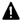
**CRITICAL STEP** Aliquoted components should be thawed right before use, repeated freeze-thaw cycles should be strictly avoided.
3. Detach C-tube from the dissociator when the program terminates. Gently resuspend obtained liver homogenate and filter it through a pre-wetted 100 µm cell strainer into a 15 mL falcon tube. To avoid loss of cells within the C-tube, wash the tube and strainer with 5 mL of cell culture medium (Figure 2F,G).
4. Centrifuge the homogenate sample for 10 min at 300× *g*, RT. Discard supernatant and resuspend the pellet in PBS (RT).

### 3.3. Liver Homogenate Processing to Prepare Single Cell Suspension

**Figure 4:**
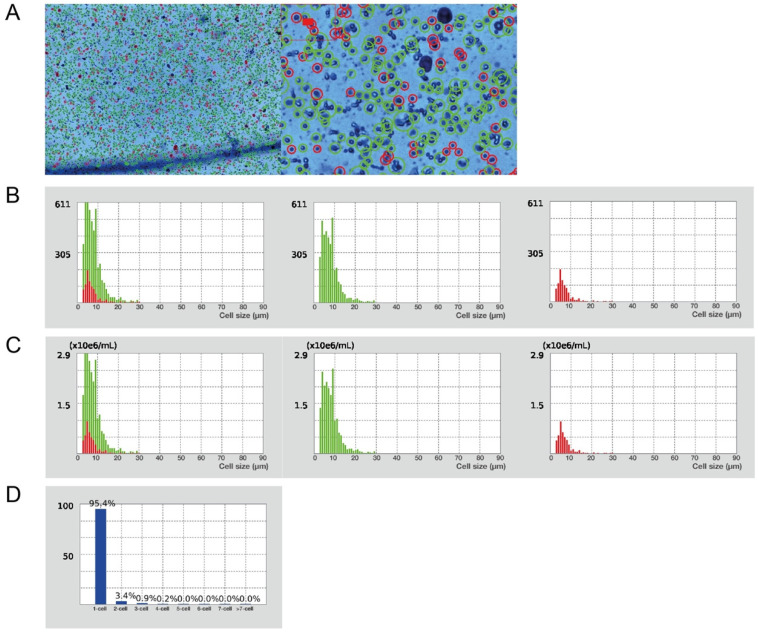
Characteristics of the obtained single cell suspension. (**A**) Evaluation of cell viability and total cell concentration (Trypan Blue stain, 1× and 4× magnification in Luna cell counter). Green circles represent live cells (82%); red circles dead cells (18%). Total yield of 2.2 × 10^7^ live cells/mL from 1 g of liver tissue. (**B**) Cell size distribution by cell number. Green histograms represent live cells; red histograms represent dead cells. (**C**) Cell size distribution by cell concentration. Green histograms—live cells; red histograms—dead cells. (**D**) Cell cluster map.

5. Centrifuge the obtained cell suspension for 10 min at 300× *g*, 4 °C and discard the supernatant.
6. Resuspend the pellet in pre-cooled PBS, add the debris removal solution and overlay gently with the pre-cooled PBS (according to the manufacturer’s instruction).
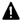
**CRITICAL STEP** Observe phase formation to control the step (Figure 2H).
7. Centrifuge for 10 min at 3000× *g*, 4 °C.
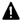
**CRITICAL STEP** Reduce the centrifugation break as well as acceleration rate (level 4 out of 9 applied on centrifuge used in this protocol), 3 phases have to be well defined.
8. Aspirate the two upper phases and add up to 15 mL of pre-cooled PBS, mix the suspension by gentle inverting the tubes.
9. Centrifuge for 10 min at 1000× *g*, 4 °C and discard the supernatant (Figure 2I).
10. Resuspend pellet in 1 mL of RBL to remove remaining red blood cells and incubate for 5 min at RT.
11. Fill the tube with PBS, mix the suspension by gentle inverting the tube.
12. Centrifuge for 5 min at 500× *g*, RT and discard the supernatant (Figure 2J).
13. **OPTIONAL STEP** Repeat the steps C.10–C.12 if pelleted cells are still contaminated with red blood cells, eventually platelets.
14. Resuspend the pellet in at least 1 mL of PBS to count the cells.
15. Use any cell counter to determine cell concentration, viability on the basis of Trypan blue exclusion, size distribution and clustering.
16. **OPTIONAL STEP** Using the LUNA cell counter, follow the steps below (17–19).
17. Prepare a 1:1 mixture of cells and Trypan blue (10 µL + 10 µL) to determine the cell count (concentration), viability, distribution, and clustering. Apply 10 µL of the mixture into a cell counting slide chamber, wait until the equilibrium is established.
18. Set the counting protocol to the following settings: dilution factor 2, min. cell size 3 µm, max. cell size 30 µm, size gating 3–30 µm, live cell sensitivity 7, roundness 60%, declustering level medium.
19. Apply the loaded protocol on a sample, verify the autofocus and count the cells, verify the gating strategy of the program (Figure 4).

### 3.4. FC Based Immunophenotyping

**Note**: Type of samples: immunophenotyping samples, unstained control, antibody isotype controls, fluorescence minus one (FMO) controls, positive control for live/dead marker (dead cells), single stained controls for compensation matrix (set up using compensation beads).

1. Centrifuge the obtained cell suspension for 10 min at 300× *g*, 4 °C and discard the supernatant. Wash with an excessive volume of PBS and centrifuge for 5 min at 500× *g*, RT. Discard the supernatant.
2. Resuspend cells in 40 µL of PBS.
3. To distinguish live and dead cells, add the live/dead Zombie NIR marker at a pre-determined dilution (Table 1) and incubate for 20 min at RT, avoid light.
  **Note:** Any other viability dye can be used. The used Zombie viability kit is a fixable (paraformaldehyde or methanol), an amine-reactive fluorescent dye that is non-permeant to live cells.
4. In parallel, prepare a positive control of dead cells by boiling 0.3–0.5 × 10^6^ cells in 40 µL PBS for 5 min at 65 °C, cool the sample down to RT, perform staining as in D.3 step. This sample is also used as a single stain control to create a compensation matrix.
5. Wash the cells by adding 150 µL of PBS (RT) and centrifuge for 5 min at 500× *g*, RT. Discard the supernatant.
6. Perform specific staining by resuspending the pellets in 40 µL of FC staining buffer (see Reagent Setup), incubate with the staining antibody mixture or relevant isotype controls at a pre-determined concentration (Table 1) for 30 min at 4 °C, avoid light.
7. Wash the cells by adding 150 µL of FC buffer and centrifuge for 5 min at 500× *g*, RT, discard the supernatant.
8. **OPTIONAL STEP** Fix the cells by resuspending the pellets in 80 µL of Fixation buffer, incubate for 15–45 min at RT, avoid light; wash the cells by adding 150 µL of FC buffer and centrifuge for 5 min at 500× *g*, RT, discard the supernatant.
9. Resuspend the cells in 250 µL of FC buffer and transfer cell suspension into a FC tube through its cell strainer snap cap. Samples without the fixation step are intended for immediate analysis; however, fixed samples can be stored at 4 °C for up to one week and then assayed.
10. For a compensation matrix set up, prepare single stained samples using a drop of both types of compensation beads (anti-rat/hamster and negative particle set, Table 1) into 30 µL of FC buffer (1 drop is approximately of 50 µL equivalent). Perform staining directly in FC tubes to minimize potential losses.
11. Add the specific staining antibody in the same dilution as for the immunophenotyping (count sample volume as a composition: 50 µL drop of specific + 50 µL negative beads + 30 µL of FC buffer), incubate under the same conditions as in step 6.
12. Wash the beads by adding 2 mL of FC buffer and centrifuge for 10 min at 200× *g*, RT, discard the supernatant.
13. Resuspend the pelleted beads in 250 µL of FC buffer, vortex thoroughly.
14. Launch a calibration procedure at the flow cytometer, create the compensation matrix using the unstained and single stained samples, and calculate the compensations.
15. **OPTIONAL STEP** If required, define the acquisition mode in terms of cells or beads used for the compensation set up depending on the flow cytometer available.
16. Formulate the gating strategy (Figure 3) to monitor cell subsets of interest, respect subset hierarchy and marker exclusivity.
17. Run samples for the immunophenotyping purposes by gating on a rare population (either KC or neutrophils).
18. Acquire and record data by collecting at least 10,000 events of the population of interest.
19. **OPTIONAL STEP** If necessary, record the same sample several times by gating on various immune populations to then easily define and characterize any population.
20. Export fsc files to evaluate the data in FlowJo software or any equivalent.

### 3.5. Data Analysis

**Figure 5:**
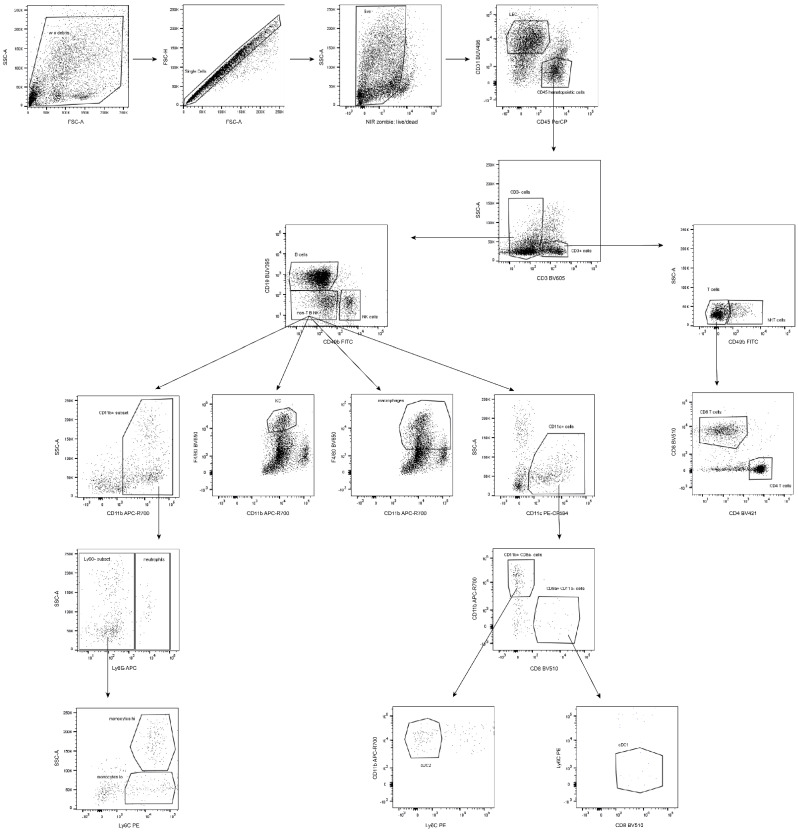
Illustration of gating strategy for individual immune populations. (Shown images represent a composition of three independent measurements).

1. Start the gating strategy first by the debris exclusion, looking at forward and side scatter, followed by a doublet and dead cell exclusion.
2. Gate the particular immune population by exclusion of non-desired cells and the selection of those specific cells (Table 2, Figure 3 and Figure 5).
  **Note:** Zombie viability dye is permeant only to cells with compromised membranes, therefore a negative population needs to be gated as live subset.
3. **OPTIONAL STEP** To quantify individual populations and their subsets, export the frequency of various subsets as a percentage in either the live cells or the parent population regarding the data representation.

## 4. Expected Results and Discussion

The key to a successful isolation and characterization of liver immune cells is an effective PBS-based liver perfusion. The perfusion via the liver portal vein can be technically challenging, considering the relative size of the vein (internal radius about 0.12 cm [31]). Here, the critical step is the selection of the correct size and length of the cannula. In this protocol, we recommend using a 24G cannula without wings and a safety lock, 19 mm length and 0.74 mm external catheter to achieve a stable and continuous perfusion. The next important step is the portal vein stretching (e.g., with forceps as shown in Figure 2A), thanks to which the vein is more visible, accessible, and easily injectable. Then, during the perfusion, the PBS flow needs to be continuous to avoid blockage in terms of bumps around the vein, liver, pancreas, stomach, and no PBS leakage should occur. The sign of correct perfusion is an immediate and gradual color change of the liver from dark red, through brown and pinkish, to beige. Overall, the liver becomes blanched and slightly swollen. In the case of limited areas of re-coloring, the cannula should be slightly moved backwards and forwards, and/or the liver can be smoothly and gently rolled over with a wet cotton pad to free a potentially blocked PBS flow.

If the protease-based perfusion of the liver alters cell surface markers on immune cells [4], a thorough optimization process would be required, unlike the simple PBS-based technique used in this protocol. The PBS-based perfusion allows removing immune cells present in blood, and it is faster and technically not as challenging as the enzymatic perfusion [22]. In addition, the PBS-based perfusion is compatible with the immunohistopathology analysis. The detailed identification and potential quantification of specific subsets relies on one in time combination of a mechanical disruption and an enzymatic digestion of the liver tissue, optimized by Miltenyi Biotec [30]. In order to avoid incomplete tissue dissociation, it is important not to exceed the maximum weight of 1.2 g of a tissue per cell isolation. Inappropriate temperature settings or an incorrect order of individual protocol steps would provide potentially misleading data. 

To obtain high quality flow cytometry data, it is also essential to remove cell debris and the remaining hepatocytes from samples, as this detritus can create an irrelevant background as well as interfering autofluorescence during the sample acquisition. For further FC staining of NPC, knowledge of the cell concentration and viability is mandatory. For this purpose, we have chosen the LUNA cell counter to simplify the workflow, to focus on reproducibility, and to determine the cluster map (Figure 4). The typical yield of NPC from one 1 g of liver tissue has been more than 20 × 10^6^ cells, yet it may differ based on the mouse strain used and the age of the mouse [14,32,33]. This protocol is optimized to produce not only a high number of cells with viability more than 80% (Figure 4A), but also to obtain a single cell suspension (more than 95% of single cells, (Figure 4D)). Nevertheless, a slow and harsh workflow and/or inappropriate cell processing can rapidly decrease the viability of cells and may also negatively affect the FC data as antigen/epitope may degrade. Unlike Medina-Montano, et al. [34], we believe that combination of liver perfusion and mechanical disruption with enzymatic digestion is mandatory to obtain exclusively hepatic immune microenvironment thus avoiding blood specific immune cell contamination. Using a multiparametric flow cytometry-based phenotyping requires a precise compensation matrix due to the high spillover signals. To determine the undesirable autofluorescent character of tissue and to correctly quantify cells, an unstained sample is required. This issue can be overcome by using spectral flow cytometer as it measures full range of emission spectrum of each fluorochrome across all lasers [27] or CyTOF-based technique which uses unique isotope-conjugated markers without need of compensation [15]. However, different sets of controls such as single stain controls need to be used. Regarding the basic immunophenotype determination as well as the particular quantification, it is crucial to define a proper gating and a non-specific antibody binding, thanks to the FMO and antibody isotype controls. The so-called isoclonic antibody control could be an alternative to the antibody isotype controls, as it is based on the staining with the excess of an identical, yet unlabeled antibody related to the specific immune marker. However, we present the use of a relevant isotype control. 

The gating strategy for the presented multicolor FC panel (Figure 3 and Figure 5) is based on the gradual elimination of unwanted populations and further identification of targeted subsets. The additional combination of immune profiling can expand the obtained datasets. The introduced FC panel (Table 1) is not limited by the determination of basic populations as it can be extended to particular (sub)phenotypes of NK or NKT subsets, e.g., cytotoxic CD8+ subpopulations. In the presented gating strategy, we show the simple approach of gating the inflammatory (or classical) Ly6C^high^ monocytes and reparative (also called non-classical or patrolling) Ly6C^low^ monocytes in order to monitor the overall changes of many immune subsets. However, recent studies describe multiple subsets of monocytes with distinct functions [35]. As such, if monocytes were the center of focus, a monocyte-specific panel should be designed. In addition, although neutrophils are characterized as Ly6G+, which is their distinguishing feature, they also express Ly6C [36]. However, the low Ly6C expression characterizes the myeloid-derived suppressor cells of neutrophil origin [37]. These alternative gating strategies can provide supplementary information on ongoing immune reactions such as inflammation, immunosuppression [38], and even tumor immune responses [39]. Accordingly, the gating of functional cells could be a useful approach in therapeutic studies (cancer, autoimmune diseases, etc.). Concerning data exportation and interpretation, the frequency of cell population in live cells can be used for monitoring the changes of immune subset ratios in various pathophysiological conditions [40]. In addition, the frequency of parent population is suitable for monitoring the expression of a particular marker within the population of interest.

In addition to the FC-based immunophenotyping, the quality control of isolated cells could be verified by functional assays. For example, in vitro lipopolysaccharide (LPS) treatment induces tumor necrosis factor alpha (TNFα) production by KC [18]. Alternatively, other functional analyses can be applied for KC, HSC or LSEC [41] or any other population of interest.

Taken all together, our complete protocol allows a highly effective and comprehensive progression in liver immune research and in the understanding of various pathologies.

## 5. Reagents Setup

1. RBL buffer
  - 0.1 mM EDTA
  - 12 mM NaHCO_3_
  - 155 mM NH_4_Cl
  - Store at RT, no contamination should appear.
2. FC staining buffer
  - 0.5% BSA (*w*/*v*)
  - 2 mM EDTA
  - Prepare a solution in 1x PBS. Store at 4 °C up to 1 month without any preservative such as 0.02% (*v*/*v*) thimerosal or 0.02–0.05% (*w*/*v*) sodium azide.

## Figures and Tables

**Table 1:** Materials for immunophenotyping.

Cell Staining (cat. no.)	Clone	Dilution	Isotype Controls (cat. no.)	Manufacturer	Staining Buffer	FC Compensations
rat anti-mouse CD3 BV605 (564009)	17A2	1/100	BV605 Rat IgG2b, κ (563145)	BD Biosciences	FC	CompBead Anti-Rat and Anti-Hamster Ig κ/Negative Control Compensation Particles Set (552845)
rat anti-mouse CD4 BV421(562891)	GK1.5	1/50	BV421 Rat IgG2b, κ (562603)	BD Biosciences	FC	
rat anti-mouse CD8 BV510 (563068)	53-6.8	1/50	BV510 Rat IgG2a, κ (562952)	BD Biosciences	FC	
rat anti-mouse CD11b APC-R700 (564985)	M1/71	1/100	APC-R700 Rat IgG2b, κ (564984)	BD Biosciences	FC	
hamster anti-mouse CD11c PE-CF594 (565591)	N418	1/50	PE-CF594 Hamster IgG2, λ1	BD Biosciences	FC	
rat anti-mouse CD19 BUV395 (563557)	1D3	1/100	BUV395 Rat IgG2a, κ (563556)	BD Biosciences	FC	
rat anti-mouse Ly-6C PE (560592)	AL-22	1/50	PE Rat IgM, κ (553943)	BD Biosciences	FC	
rat anti-mouse Ly-6G APC (560599)	1A8	1/50	APC Rat IgG2a κ (553932)	BD Biosciences	FC	
rat anti-mouse CD45 PerCP (561047)	30-F11	1/100	PerCP Rat IgG2b, κ (552991)	BD Biosciences	FC	
rat anti-mouse CD49b FITC (553857)	DX5	1/100	FITC Rat IgM, κ (553942)	BD Biosciences	FC	
rat anti-mouse CD31 BUV496 (741084)	390	1/100	BUV496 Rat IgG2a, κ (564663)	BD Biosciences	FC	
rat anti-mouse F4/80 BV650 (743282)	T45-2342	1/50	BV650 Rat IgG2a, κ (563236)	BD Biosciences	FC	
live/dead marker Zombie NIR (423106)	n/a *	1/200	n/a *	Biolegend	PBS	cells

NOTE: working concentrations of either FC antibodies or related isotype controls were identical. * n/a: not applicable.

**Table 2:** Phenotypes of particular immune populations.

Immune Population	Immunophenotype
liver endothelial cells	CD31+ CD45−
hematopoietic cells (leukocytes)	CD31− CD45+
T cells	CD31− CD45+ CD3+ CD49b−
helper T cells	CD31− CD45+ CD3+ CD49b− CD4+ CD8−
cytotoxic T cells	CD31− CD45+ CD3+ CD49b− CD4− CD8+
B cells	CD31− CD45+ CD3− CD49b− CD19+
NK cells	CD31− CD45+ CD3− CD49b+
NKT cells	CD31− CD45+ CD3+ CD49b+
neutrophils	CD31− CD45+ CD3− CD19− CD49b− CD11b+ Ly6G+
reparative monocytes	CD31− CD45+ CD3− CD19− CD49b− CD11b+ Ly6G− Ly6C^lo^
inflammatory monocytes	CD31− CD45+ CD3− CD19− CD49b− CD11b+ Ly6G− Ly6C^hi^
CD8 cDC1	CD31− CD45+ CD3− CD19− CD49b− CD11c+ CD8+ CD11b− Ly6C−
CD11b cDC2	CD31− CD45+ CD3− CD19− CD49b− CD11c+ CD8− CD11b+ Ly6C−
KC	CD31− CD45+ CD3− CD19− CD49b− CD11blo F4/80hi
macrophages	CD31− CD45+ CD3− CD19− CD49b− CD11b+ F4/80+

## Data Availability

Not applicable.
